# CBIcall: a configuration-driven framework for variant calling in large sequencing cohorts

**DOI:** 10.1093/bioadv/vbag232

**Published:** 2026-08-12

**Authors:** Manuel Rueda, Dietmar Fernandez-Orth, Ivo G Gut

**Affiliations:** Centro Nacional de Análisis Genómico, Barcelona 08028, Spain; Universitat de Barcelona (UB), Barcelona, Spain; Centro Nacional de Análisis Genómico, Barcelona 08028, Spain; Universitat de Barcelona (UB), Barcelona, Spain; Centro Nacional de Análisis Genómico, Barcelona 08028, Spain; Universitat de Barcelona (UB), Barcelona, Spain

## Abstract

**Motivation:**

Variant calling for next-generation sequencing (NGS) data relies on a diverse ecosystem of tools and workflows. Large-scale collaborative studies increasingly adopt federated analysis, where each institution processes sensitive data locally using standardized pipelines. Deploying identical pipelines across multiple centers remains challenging because heterogeneous software environments and computing policies can cause workflow divergence and inconsistent results.

**Results:**

We developed CBIcall, a workflow backend-flexible, configuration-driven framework that runs standardized variant-calling pipelines from raw FASTQ files to analysis-ready VCFs. Users define each analysis in a single YAML parameters file, which CBIcall resolves against a controlled workflow registry and resource catalog. The execution driver validates parameters and checks compatibility among pipelines, analysis modes, workflow backends, genome builds, tool versions, and resource bundles. CBIcall supports reproducibility auditing by comparing executions using recorded provenance and output fingerprints. CBIcall dispatches validated workflows natively through Bash, Cromwell, Nextflow and Snakemake backends and provides production-ready pipelines for germline WES, WGS (single-sample or cohort joint genotyping following GATK Best Practices), and mitochondrial DNA analysis. We evaluated analytical performance using public benchmark datasets and validated reproducibility across four computing environments. We further deployed CBIcall in the EU HEREDITARY project, where it processed 1102 samples with both WES and mtDNA pipelines on an institutional HPC system, supporting its suitability for reproducible cohort-scale genomic analyses.

**Availability and implementation:**

CBIcall is open source (GPLv3) and distributed with ready-to-run pipelines; full dependency and installation documentation is available at https://github.com/CNAG-Biomedical-Informatics/cbicall.

## 1 Introduction

Next-generation sequencing (NGS), introduced in 2005, has transformed genomic research by making DNA and RNA profiling scalable and cost-efficient (Metzker 2010, Goodwin *et al*. 2016). As sequencing technologies have matured, the software ecosystem has evolved from simple command-line utilities to complex workflow engines and multi-stage analysis (Köster and Rahmann 2012, Amstutz *et al*. 2016, Di Tommaso *et al*. 2017, Voss *et al*. 2017, Ewels *et al*. 2020, Galaxy Community 2024). This diversity provides analytical flexibility, but it also creates practical challenges for collaborative studies that must analyze large cohorts using stable, consistent methods across sites.

Many European research projects now use federated analysis models, where each institution processes genomic data locally to comply with legal and ethical requirements (Rehm *et al*. 2021). A key challenge is executing identical, validated pipelines across heterogeneous HPC environments that differ in software stacks, scheduling policies, and file system conventions. Although many publicly available workflows are analytically robust, they are frequently distributed as workflow templates without a standardized validation layer that enforces configuration correctness, compatible tool versions, and consistent runtime environments. As a result, deployments across different institutions often require site-specific wrappers and manual adjustments, which may introduce workflow divergence and compromise reproducibility (Leipzig 2017, Grüning *et al*. 2018, Ahmed *et al*. 2021).

To address these challenges, we developed CBIcall within the EU Horizon Europe HEREDITARY project (https://hereditary-project.eu). The framework was developed in the context of large collaborative sequencing projects at the Centro Nacional de Análisis Genómico (CNAG), where robust and reproducible execution of standardized pipelines across heterogeneous computing environments is required.

Workflow engines such as Nextflow, Snakemake, and Cromwell provide powerful execution models, while curated workflow collections offer maintained pipeline implementations. CBIcall addresses a complementary layer. Rather than introducing a new workflow engine, CBIcall provides a configuration-driven validation, execution, and audit layer that orchestrates approved workflows through supported execution backends. CBIcall currently supports Bash (Ramey and Fox 2025), Cromwell (Voss *et al*. 2017), Nextflow (Di Tommaso *et al*. 2017), and Snakemake (Köster and Rahmann 2012).

## 2 Methods

### 2.1 Overall design and system requirements

CBIcall is a configuration-driven framework designed to ensure consistent and reproducible variant-calling workflows across institutional computing environments, including high-performance computing (HPC) clusters commonly used in large-scale sequencing centers. The system provides ready-to-use pipelines for nuclear and mitochondrial DNA variant calling, processing paired-end FASTQ files into VCF files.

CBIcall adopts a three-layer execution contract:

1. *User parameters YAML:* specifies the analysis intent, including input samples, pipeline and mode selection, genome build, workflow backend (Bash, Cromwell, Nextflow and Snakemake) and tool parameters (example provided in Supplementary Text S1, available at supplementary material  *Bioinformatics Advances* online).
2. *Workflow registry YAML:* maps supported workflow providers (e.g., cbicall-core and nf-core), pipelines and workflow backends to approved executable workflow definitions (example provided in Supplementary Text S2, available at supplementary material  *Bioinformatics Advances* online).
3. *Resource catalog:* defines the external resource layer, including reference bundles, compatible workflows, resource identity and integrity metadata (example provided in Supplementary Text S3, available at supplementary material  *Bioinformatics Advances* online).

### 2.2 Execution control layer

**Figure 1:**
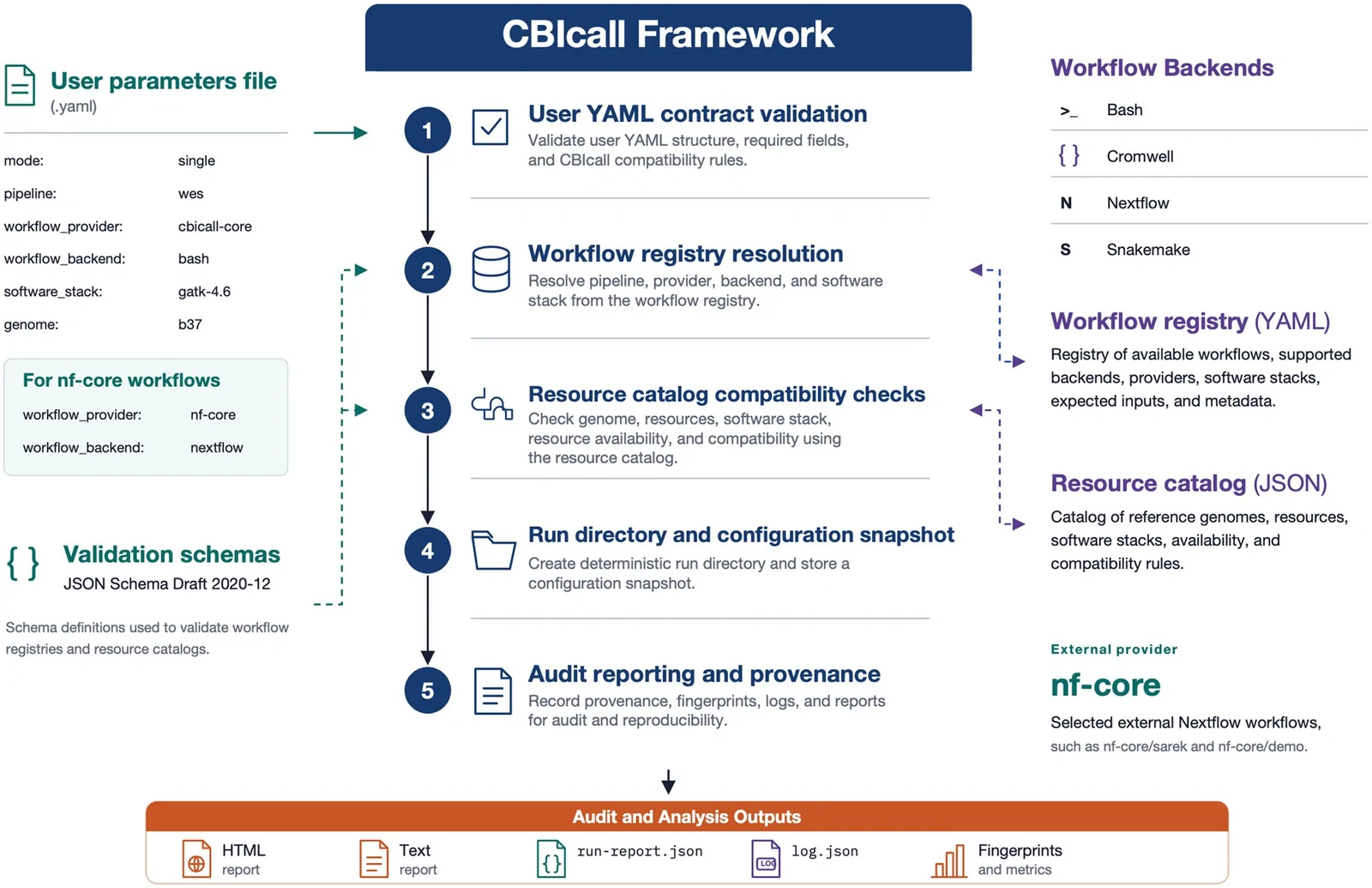
Overview of the CBIcall execution model. Analyses are defined through a user YAML configuration. The configuration is validated against an internal workflow registry and resource catalog before workflow execution. This multi-layer validation model ensures consistency between the requested analysis, workflow implementation, and resource bundle.

The CBIcall driver (implemented in Python 3) serves as the framework’s central execution controller (Fig. 1). For each run, the driver loads the user’s parameters YAML and validates it against controlled vocabularies and compatibility rules. These checks cover analysis mode, genome build, workflow backend, workflow provider, software stack (including specific version constraints for GATK (McKenna *et al*. 2010)), and the selected resource catalog entry; resolved tool versions are recorded in the run audit when available. The CBIcall driver introduces minimal computational overhead (27 MiB RAM), while computational resource requirements are determined primarily by the underlying workflow backend and external bioinformatics tools.

Workflow registry and resource catalog definitions are validated using JSON Schema Draft 2020-12. After validation, the system creates a standardized run directory and launches the selected workflow through the configured backend. Validation and execution control remain implemented in the core framework, while backend-specific workflow code is kept in the registered workflow definitions. This validation model enables CBIcall to execute curated nuclear and mitochondrial variant-calling workflows while ensuring compatibility among the selected analysis mode, workflow backend, workflow provider, genome build, software versions, and resource bundle before execution.

Table 1 places CBIcall within the existing workflow ecosystem, highlighting its complementary role relative to workflow engines and curated workflow collections.

**Table 1:** Conceptual positioning of CBIcall relative to workflow engines and curated workflow collections.

Scope	Workflow engines	Curated workflow collections	CBIcall
Primary role	Execute workflow logic	Provide maintained workflow implementations	Validate, orchestrate and audit configured analyses
Examples	Nextflow, Snakemake, Cromwell	nf-core/Sarek, nf-core/demo	Native (cbicall-core) and external-provider workflows
User configuration	Engine- or pipeline-specific parameters	Pipeline-specific parameters	Single validated YAML configuration
Workflow implementation	Defined by workflow authors	Maintained by pipeline developers	Native or approved external workflows
Resource management	User- or pipeline-dependent	Pipeline-dependent	Central validated resource catalog
Execution model	Engine-specific	Pipeline-specific	Common execution contract across supported backends
Provenance	Engine-generated logs/reports	Pipeline-generated reports	Structured provenance, fingerprinting and run comparison

### 2.3 Run audit and reproducibility reporting

Each CBIcall execution produces structured provenance and audit artifacts, including log.json, run-report.json, and an optional static HTML report. These files capture the resolved parameters, software versions, runtime context, workflow fingerprints, resource identity, output inventories, normalized VCF hashes, and task-level provenance when available. Similar provenance-capture approaches have been proposed to support workflow interoperability and reproducibility tracking, including Workflow Run RO-Crate (Leo *et al*. 2024).

CBIcall also provides a compare-runs command that uses recorded audit artifacts to compare two or more completed executions and generates text and static HTML reports. Both single-run and run-comparison summaries can optionally be exported as MultiQC custom-content files, enabling CBIcall audit summaries to be viewed alongside other QC outputs (Ewels *et al*. 2016). Related work has explored provenance differencing and reproducibility assessment through comparison of workflow executions and outputs (Missier *et al*. 2016, Suetake *et al*. 2022). In contrast, CBIcall focuses on operational auditing and reproducibility governance by comparing workflow identities, resource identities, execution metadata, output inventories, and final-output fingerprints.

### 2.4 Workflow backends and validated pipelines

CBIcall currently natively supports four workflow backends: Bash, Cromwell, Nextflow and Snakemake. The Bash backend provides ready-to-use pipelines for whole-exome sequencing (WES), whole-genome sequencing (WGS), and mitochondrial DNA (mtDNA) variant calling. Legacy Bash pipelines use GATK 3.5 (McKenna *et al*. 2010) for WES and mtDNA analyses (Rueda *et al*. 2017), whereas current nuclear WES and WGS use GATK 4.6 (Poplin *et al*. 2018). For WES analyses, interval definitions are tied to the selected workflow resources: the legacy GATK 3.5 WES workflow uses Agilent SureSelect target intervals, while the GATK 4.6 WES workflow uses the Broad exome interval list distributed with the GATK b37 resource bundle. Nuclear germline pipelines follow GATK Best Practices (DePristo *et al*. 2011, Van der Auwera *et al*. 2013) and include read alignment with BWA-MEM (Li 2013), coordinate sorting, duplicate marking, base quality score recalibration, per-sample gVCF generation using *HaplotypeCaller*, cohort-level genotyping with *GenotypeGVCFs*, variant quality score recalibration, and standard post-processing filters (see online documentation).

For mitochondrial DNA, variant calling uses MToolBox (which requires GATK 3.5) (Calabrese *et al*. 2014). Mitochondrial DNA analyses produce a VCF file and a prioritized annotation file, from which CBIcall creates an interactive HTML report (see Supplementary Fig. S6, available at supplementary material  *Bioinformatics Advances* online) adapted from a previously described framework (Rueda and Torkamani 2017). For WES and WGS analyses, the VCFs created with CBIcall can be integrated with external downstream tools, such as *beacon2-cbi-tools* (formerly *beacon2-ri-tools*) for variation annotation and Beacon v2 API data exchange format conversion (Rueda *et al*. 2022).

The GATK 4.6 WES and WGS workflows are also implemented as native CBIcall workflows for Cromwell, Nextflow, and Snakemake. These implementations follow the same CBIcall execution contract as the Bash backend, including parameter validation, deterministic run directories, structured logging, provenance capture, and final-output reporting. Thus, CBIcall can execute the same validated analysis logic through different workflow backends while preserving a common audit and reproducibility layer.

Workflow implementations from community repositories such as WorkflowHub (Gustafsson *et al*. 2025) or Dockstore (Yuen *et al*. 2021) can, in principle, be adapted and registered through the CBIcall YAML workflow registry without modifying the core framework. When adapted to conform to the CBIcall output contract—including the expected run-directory layout, logs, final outputs, and reportable deliverables—they are treated as CBIcall-native workflows (see developer documentation at https://cnag-biomedical-informatics.github.io/cbicall/). Workflows that retain their original upstream output structure instead require explicit external-provider support, allowing CBIcall to generate the appropriate backend-specific launch configuration, identify canonical outputs, and audit the resulting execution.

In the current implementation, external-provider support is available for selected nf-core workflows (Ewels *et al*. 2020), specifically nf-core/sarek (Hanssen *et al*. 2024) and nf-core/demo (Hakkaart *et al.* 2025), using Nextflow as the execution backend. In this mode, the upstream nf-core project remains responsible for the workflow implementation, dependency profiles, execution logic, and pipeline-specific outputs. CBIcall provides an additional governance layer that validates workflow selection, user parameters, workflow provider, execution backend, resource configuration, and runtime options before execution. After completion, runs are processed through the same audit framework used for native workflows, generating structured reports that record workflow and resource identities, execution metadata, output inventories, and, when configured, cryptographic fingerprints of the final outputs. This allows selected community workflows to execute under the same CBIcall reproducibility and traceability framework as native workflows without requiring modifications to the upstream pipeline code.

### 2.5 Containerization and portability

CBIcall is distributed as a container image that includes the execution framework and its Python dependencies, and it can alternatively be installed from source. External resources—including reference genomes, annotation databases, and third-party tools such as GATK, BWA-MEM, and Samtools (see list at Supplementary Table S1, available at supplementary material  *Bioinformatics Advances* online)—are distributed separately and must be downloaded and placed in a designated resource directory as described in the online documentation. Native CBIcall resource bundles can be installed automatically using the framework installation utility, which downloads or accepts the registered bundle files, verifies the catalog-declared archive or split-part checksums, prepares the local resource directory, and validates resource identity and workflow compatibility against the resource catalog before execution.

In containerized environments, users mount this external resource directory into the container and register it through a configuration file. On institutional HPC systems that do not support Docker, the same container image can be executed using Apptainer (formerly Singularity), enabling consistent deployment across heterogeneous computing environments.

## 3 Results: validation and use cases

### 3.1 Validation of the CBIcall framework

#### 3.1.1 Accuracy assessment using GIAB benchmark dataset

To evaluate the analytical performance of native workflows executed through CBIcall, we benchmarked the CBIcall WGS pipeline using the Ashkenazim trio from the Genome in a Bottle (GIAB) consortium (Zook *et al*. 2019), consisting of HG002 (son), HG003 (father) and HG004 (mother) reference samples. The original high-coverage sequencing data (300×) were downsampled to 10% using seqtk (Li n.d.), which performs random read subsampling by retaining each read with an independent probability of 0.1. Sequence reads were processed with the CBIcall WGS single-sample workflow using GRCh38, and the resulting gVCFs were subsequently processed with the CBIcall WGS cohort workflow for joint genotyping.

The final recalibrated BAM files showed mean genome-wide coverages of 30.5×, 31.0×, and 31.8×, respectively. Variant calling performance was assessed against the GIAB v4.2.1 benchmark set for GRCh38 using hap.py (vcfeval engine) following GA4GH benchmarking best practices (Krusche *et al*. 2019). Evaluation was restricted to GIAB high-confidence regions and included standard metrics for SNVs and small indels, including precision, recall, and F1 score.

Across the GIAB trio, SNV F1 scores were 99.12%–99.15% for all calls and 94.91%–94.93% for PASS calls; indel F1 scores were 98.52%–98.60% for all calls and 95.39%–95.71% for PASS calls (Table S2, available at supplementary material  *Bioinformatics Advances* online). These results indicate that workflows executed through CBIcall produce variant call sets consistent with established community reference standards (Zhao *et al*. 2020). The benchmark can be reproduced using the commands and input preparation steps provided in the CBIcall online documentation.

#### 3.1.2 Reproducibility across heterogeneous computing environments

To evaluate the portability and reproducibility of CBIcall, a WES sample from the 1000 Genomes Project, HG00103 (SRR1596639), was processed using identical workflow definitions across four distinct computational environments: (i) Google Cloud Platform with VM Ubuntu 22.04, (ii) an x86_64 workstation with Linux Mint 20.3, (iii) a macOS ARM64 workstation with VM Ubuntu 24.04, and (iv) a SLURM-managed HPC cluster running x86_64 CentOS Linux release 7.9.2009.

All executions were performed using the same CBIcall YAML contract, workflow definition, software stack, and resource bundle. Completed runs were compared using cbicall compare-runs, which evaluates workflow identity, resource identity, execution metadata, output inventories, and normalized VCF fingerprints. Across the four computational environments, the final VCF contained 23 562 records, including 19 578 PASS and 3984 non-PASS records. The call-level VCF fingerprint, based on CHROM, POS, REF, ALT, FILTER, and sample genotype (GT) fields, was identical across all environments. A stricter full-record VCF fingerprint detected a minor numerical difference in one non-call field on the ARM-based run, without changing the reported variant call. These results demonstrate reproducible final variant calls across operating systems, processor architectures, cloud and on-premises deployments, and HPC scheduling infrastructure. Extended information is provided in the online CBIcall documentation and Supplementary Text S6, available at supplementary material  *Bioinformatics Advances* online.

### 3.2 HEREDITARY cohort use case

To illustrate the robustness of the framework in a collaborative cohort setting such as that of HEREDITARY, we applied CBIcall to two representative use cases using real sequencing datasets processed within an institutional (CNAG) HPC environment. We initially analyzed 618 WES samples from the National Institute of Neurological Disorders and Stroke (NINDS) Parkinson’s Disease study (dbGaP accession phs001172.v1.p2). Following quality control and population structure assessment, 19 samples were excluded to reduce population heterogeneity (see Supplementary Figure S1, available at supplementary material  *Bioinformatics Advances* online), resulting in a final dataset of 599 Parkinson’s disease samples from participants self-reported as North American Caucasian. Because this dataset did not include matched neurologically normal controls, it was analyzed together with 503 European-ancestry samples from the 1000 Genomes (1000G) Project, a predominantly WGS resource that also includes WES data (Genomes Project *et al*. 2015). These samples served as an external reference population, forming a combined study cohort of 1102 samples. Both nuclear and mitochondrial analyses described below were performed on this same WES-derived cohort. In the figures, the 503 European-ancestry 1000G reference samples are labeled “Controls.”

#### 3.2.1 Use case 1: nuclear variant calling in a large WES cohort

To demonstrate large-scale nuclear variant calling in this cohort, starting from FASTQ files, we processed all samples using the CBIcall WES pipeline with the b37 (hg19) reference genome (selected for compatibility with validated GATK resource bundles), followed by standard *bcftools* (Danecek *et al*. 2021) post-filtering (see Supplementary Text S7, available at supplementary material  *Bioinformatics Advances* online). This produced two QC-matched callsets from samples that differed primarily in their experimental laboratory conditions while sharing identical alignment and variant calling, enabling a direct comparison of single-sample and cohort-level calling approaches:

- Single-sample mode: We called each sample individually using GATK *HaplotypeCaller*, producing per-sample gVCF and filtered VCF files. We then merged the VCFs without cohort-level re-genotyping (see Supplementary Text S7, available at supplementary material  *Bioinformatics Advances* online for bcftools commands).
- Cohort-joint genotyping mode: The 1102 gVCF files generated in single-sample mode were combined and jointly genotyped using GATK *GenomicsDB* + *GenotypeGVCFs*. The resulting VCF was then subjected to the same filtering procedure applied in the single-sample analysis (Supplementary Text S7, available at supplementary material  *Bioinformatics Advances* online).

**Figure 2:**
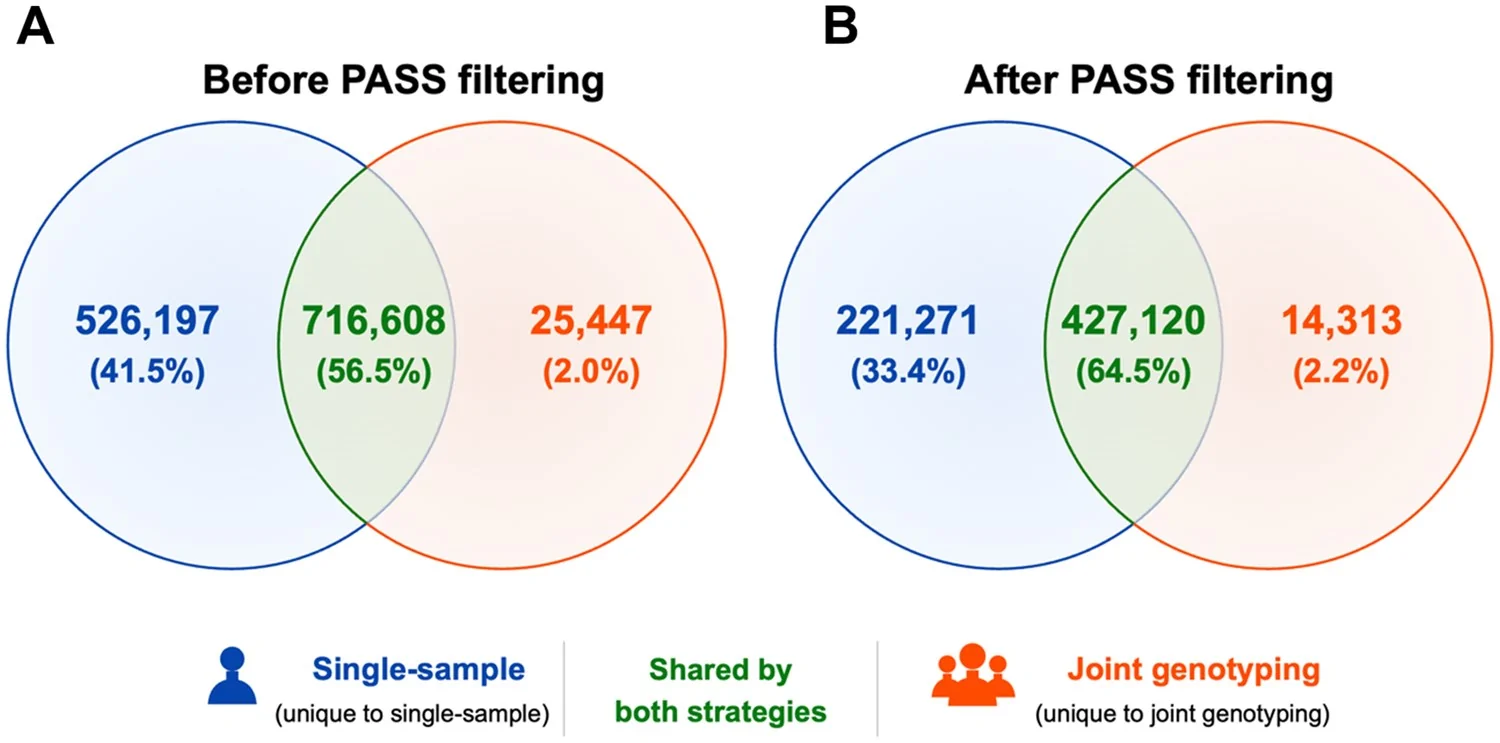
Configuration-consistent comparison of genotyping strategies using CBIcall. Venn diagram showing the overlap between variant-site level sets obtained from single-sample variant calling (followed by VCF merging), and from cohort-level joint genotyping of the same 1102 samples. (A) Variants obtained directly from calling. (B) Variants remaining after PASS filter.

As can be seen in Fig. 2A, there was a strong intersection of variants in the VCF coming from single-sample mode and the cohort-genotyping mode. After applying a PASS filter (Fig. 2B), the joint-genotyped callset retained more variants that were filtered out in the merged single-sample callset, consistent with the expected advantages of cohort-level joint genotyping (see also Supplementary Table S3, available at supplementary material  *Bioinformatics Advances* online).

The comparability of the phs001172 and 1000G cohorts was evaluated using per-sample variant counts (see Supplementary Table S4, available at supplementary material  *Bioinformatics Advances* online) and variant-site depth (DP; see Supplementary Fig. S3, available at supplementary material  *Bioinformatics Advances* online) as quality control metrics to identify potential technical biases prior to integration. Distributions of WES-derived SNVs and INDELs were highly similar across cohorts, as well as other reported metrics such as Ti/Tv (Supplementary Tables S4 and S5, available at supplementary material  *Bioinformatics Advances* online). Variant counts obtained under the two genotyping strategies were consistent, indicating no systematic shift in variant detection. Likewise, DP distributions were broadly comparable between cases and controls within each nuclear genotyping mode and within the mtDNA analysis, providing no evidence of major cohort-specific or mode-specific differences in this demonstration dataset.

Finally, we performed principal component analysis (PCA) using sample genotypes to assess population structure and potential technical bias. Cases and controls displayed largely overlapping distributions across the first two principal components, with no evident group separation (Supplementary Fig. S2, available at supplementary material  *Bioinformatics Advances* online). This analysis did not identify strong cohort-specific clustering and supports the use of the combined dataset as a representative deployment example for the CBIcall framework. As discussed above, the study was designed to demonstrate the application of CBIcall to real-world datasets rather than to perform an association analysis. Although the cohorts originate from independent studies, all samples were reprocessed from the original FASTQ files using a common workflow, ensuring consistent processing throughout the analysis. Nevertheless, the use of an external reference cohort may introduce residual differences related to cohort composition or sequencing protocols that cannot be fully avoided.

#### 3.2.2 Use case 2: mitochondrial variant calling from WES data

In this second use case, mitochondrial genome analysis was performed on the combined study cohort (1102 WES samples) using MToolBox as part of the CBIcall analysis workflow. This analysis is presented as a representative use case demonstrating the application of CBIcall to large-scale mitochondrial variant-calling workflows, rather than as a benchmark of mitochondrial variant-calling methodology. Variant calling was successful in 1037 of 1102 samples (94.1%). Overall, WES provided sufficient mtDNA variant-call depth (DP) for most samples (see Supplementary Figure S3, available at supplementary material  *Bioinformatics Advances* online). Depth was below the MToolBox threshold required for reliable variant calling in 10 of 503 1000G samples (2.0%) and 55 of 599 dbGaP samples (9.2%). After excluding these samples, 493 1000G and 544 dbGaP samples were retained for the mitochondrial analyses shown in the figures. This reflects the expected off-target nature of mitochondrial read capture in WES (Calabrese *et al*. 2014, Griffin *et al*. 2014, Poole *et al*. 2021).

**Figure 3:**
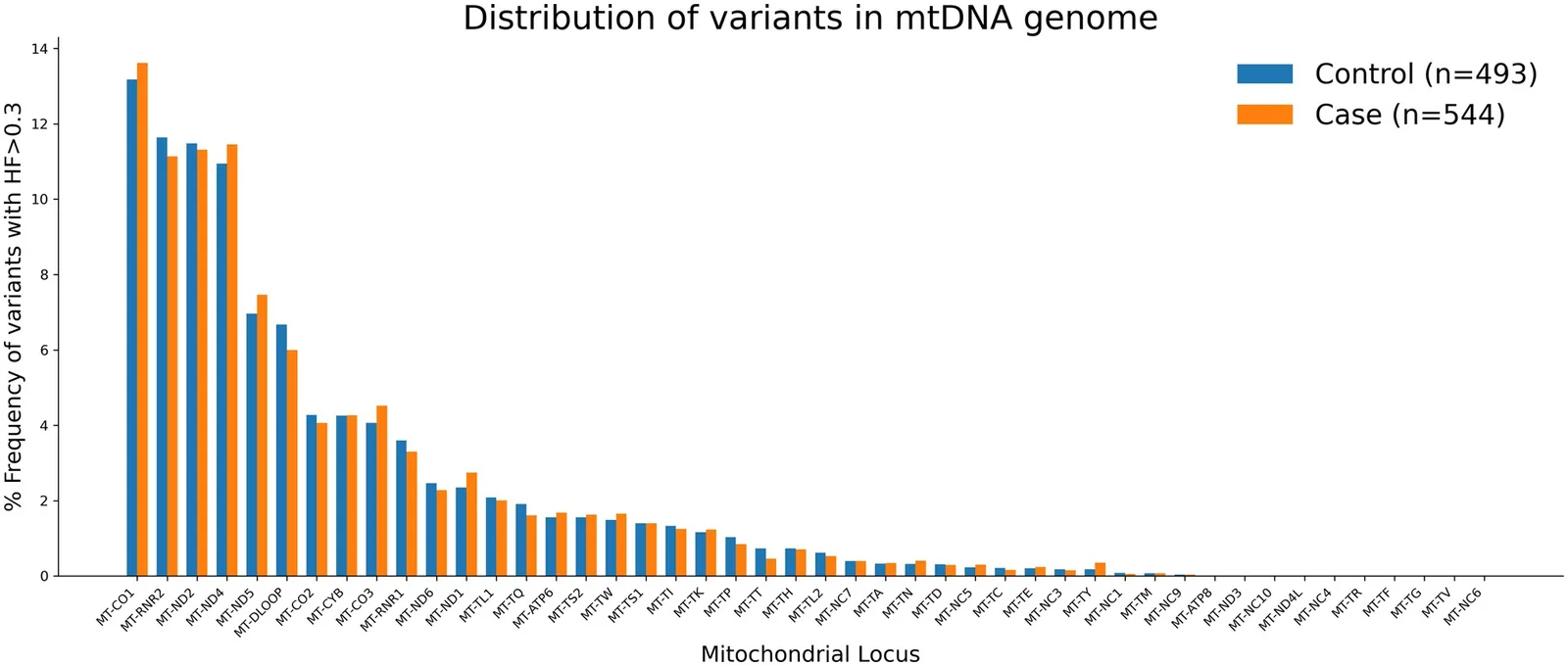
Bar plot showing the distribution of heteroplasmic variants across the 1037 samples retained for mitochondrial analysis. Note that mitochondrial loci were sorted in descending order according to the number of variants.

We performed two analyses on the annotated variants. First, heteroplasmic variants (defined as DP ≥ 25 and heteroplasmic fraction > 0.3; see Fig. S4, available at supplementary material  *Bioinformatics Advances* online) were grouped by genomic locus, and their cohort-specific and relative frequencies (as percentages) were assessed for cases and controls (Fig. 3). The results show that despite differences in sequencing strategies between the two source cohorts, the distributions of heteroplasmic variants across loci were broadly comparable between cohorts (Diroma *et al*. 2014, Rueda and Torkamani 2017, Laricchia *et al*. 2022).

Finally, to assess whether heteroplasmic variants were uniformly distributed across the mitochondrial genome, we generated a scatterplot in which loci were ordered according to their length (bp). As expected, the number of heteroplasmic variants per locus showed a consistent increasing trend with locus size in both case and control cohorts (see also Supplementary Fig. S5, available at supplementary material  *Bioinformatics Advances* online) (Diroma *et al*. 2014, Rueda and Torkamani 2017, Laricchia *et al*. 2022).

## Supplementary Material

vbag232_Supplementary_Data

## Data Availability

The data underlying this article are available from the 1000 Genomes project at https://www.internationalgenome.org/data-portal/data-collection/phase-3 and dbGaP at https://dbgap.ncbi.nlm.nih.gov/beta/study/phs001172.v1.p2/.
