# Supplementary material for: CBIcall: a configuration-driven framework for variant calling in large sequencing cohorts
Source: Bioinform Adv. 2026 Aug 12;6(1):vbag232. doi: 10.1093/bioadv/vbag232 (PMC13549384; doi:10.1093/bioadv/vbag232)
Supplement: Supplementary file 1 [file vbag232_supplementary_data.pdf]

Supplementary Data

CBIcall: a configuration-driven framework for variant calling in large sequencing cohorts

Manuel Rueda[1,2,†,*], Dietmar Fernandez-Orth[1,2,†] and Ivo G. Gut[1,2]

[1]Centro Nacional de Análisis Genómico, C/Baldiri Reixac 4, 08028, Barcelona, Spain
[2]Universitat de Barcelona (UB), Barcelona, Spain
[*]Corresponding author. Centro Nacional de Análisis Genómico, C/Baldiri Reixac 4, 08028, Barcelona, Spain.
[†]These authors contributed equally to this work.

Supplementary Data Contents:

Supplementary Methods: CBIcall framework
Text S1: Example CBIcall user configuration file (YAML) 2
Text S2: Workflow registry excerpt 3
Text S3: Resource catalog excerpt 4
Text S4: Audit and provenance records 6
Table S1: Resource bundle contents 8

Supplementary Results: CBIcall framework validation
Table S2: Benchmark against GIAB truth set 9
Text S6: Cross-environment reproducibility assessment 10

Supplementary Results: Nuclear WES cohort use case
Text S7: Variant filtering 11
Figure S1: dbGaP samples selection 12
Figure S2: PCA Controls vs. cases 15
Figure S3: Distribution of variant-site depth across analysis strategies 17
Table S3: Variant filtering statistics for single-sample and joint genotyping analyses 18
Table S4: Summary statistics of variant counts 19
Table S5: Transition/transversion (Ti/Tv) and heterozygous/homozygous (Het/Hom) ratios for the single-sample and joint-genotyping callsets 21

Supplementary Results: Mitochondrial DNA use case
Figure S4: Mitochondrial variant calling and filtering 22
Figure S5: Relationship between mitochondrial locus length and heteroplasmic variant frequency 23
Figure S6: Example mtDNA HTML report 23

References 25

Text S1: Example CBIcall user configuration

This example YAML file illustrates a typical user configuration used to run a whole-exome sequencing (WES) analysis with CBIcall. The configuration specifies the mode ("single" or "cohort"), selected analysis pipeline, workflow backend, software stack, input directory, genome build, and other analysis parameters.

Example configuration excerpt:

```
---
mode:             single
pipeline:         wes
workflow_backend: bash
software_stack:   gatk-4.6
input_dir:        CNAG999_exome/CNAG99901P_ex
resource:         cbicall-germline-resources-v1
genome:           b37
cleanup_bam:      false
```

Text S2: Workflow registry excerpt

This excerpt from the CBIcall workflow registry illustrates how pipeline definitions are resolved into backend-specific executable workflows. The registry maps analytical pipelines defined in the user configuration to implementation-specific scripts (e.g., Bash workflows), while preserving a stable user-facing configuration interface.

Example registry structure:

```
---
workflows:
  bash:
    base_dir: "workflows/bash"
    software_stacks:
      gatk-4.6:
        helpers:
          env: "env.sh"
          coverage: "coverage.sh"
          jaccard: "jaccard.sh"
          vcf2sex: "vcf2sex.sh"
          vcf2hash: "vcf2hash.sh"
        profiles:
          cnag-hpc:
            env: "cnag-hpc-env.sh"
        pipelines:
          wes:
            single:
              default_registry_version: "v1"
              registry_versions:
                v1:
                  script: "wes_single.sh"
            cohort:
              default_registry_version: "v1"
              registry_versions:
                v1:
                  script: "wes_cohort.sh"
          wgs:
            single:
              default_registry_version: "v1"
              registry_versions:
                v1:
                  script: "wgs_single.sh"
            cohort:
              default_registry_version: "v1"
              registry_versions:
                v1:
                  script: "wgs_cohort.sh"
```

Text S3: Resource catalog excerpt

This excerpt from the CBIcall resource catalog illustrates how external dependencies are defined and validated independently from workflow code. The catalog maps resource bundles to compatible pipelines, genome builds, software stacks, and required reference files, while recording resource identity and integrity metadata. This allows CBIcall to verify that a selected analysis is executed with an approved and reproducible resource environment.


```
{
  "schema_version": 1,
  "resources": {
    "cbicall-germline-resources-v1": {
      "type": "bundle",
      "version": "v1",
      "description": "Default CBIcall external tools and reference resources",
      "compatible_workflows": [
        "bash/wes/single/gatk-3.5/v1",
        "bash/wes/cohort/gatk-3.5/v1",
        "bash/wes/single/gatk-4.6/v1",
        …
      ],
      "remote_identifier": {
        "filename": "cbicall-resource-id.json",
        "google_drive_file_id": "1Yi-LXJ2bFrpogY-Nc5Ie5iv6NrvMjeho",
        "sha256": "2ca48965a3b82a411f730769ddf020b671f08cf2b0c14ce58ec1e1d7ffce377e",
        "expected": {
          "resource_key": "cbicall-germline-resources-v1"
        }
      },
      "source": {
        "provider": "google_drive",
        "folder": "https://drive.google.com/drive/folders/13MqZk0MHN_MQdNyXwjz_QTjbl2Najkeg",
        "files": {
          "data.tar.gz.md5": "1MmeppkZ1xR9ODsBLuDWUh-2ob98Prxq6",
          "data.tar.gz.part-00": "12HQw_duxmlcASh7Yw8yL2-f-hyjqMim9",
          …
        }
      },
      "archive": {
        "source_name": "data.tar.gz",
        "canonical_name": "cbicall-germline-resources-v1.tar.gz",
        "checksum_file": "data.tar.gz.md5",
        "checksum_algorithm": "md5",
        "parts": [
          "data.tar.gz.part-00",
          …
        ]
      },
      "layout": {
        "datadir": ".",
        "dbdir": "Databases",
        "ngsutils": "NGSutils",
        "expected_top_level": [
          "Databases",
          "NGSutils"
        ]
      },
      "tools": {
        "gatk4": {
          "version": "4.6.2.0",
          "path_hint": "NGSutils/gatk/gatk-4.6.2.0/gatk"
        },
        "bwa": {
```

```
          "version": "0.7.18",
          "path_hint": "NGSutils/bwa-0.7.18/bwa"
        },
        "samtools": {
          "version": "0.1.19",
          "path_hint": "NGSutils/samtools-0.1.19/samtools"
        }
      },
      "resource_sets": {
        "b37": {
          "reference_fasta_hint":
"Databases/GATK_bundle/b37/references_b37_Homo_sapiens_assembly19.fasta",
          "known_sites_hint": [
            "Databases/dbSNP/human_9606_b144_GRCh37p13/All_20160408.vcf.gz",
            "Databases/GATK_bundle/b37/b37_Mills_and_1000G_gold_standard.indels.b37.vcf.gz"
          ]
        },
        "hg38": {
          "reference_fasta_hint":
"Databases/GATK_bundle/hg38/resources_broad_hg38_v0_Homo_sapiens_assembly38.fasta"
        },
        "rsrs": {
          "description": "Mitochondrial resources used by MToolBox workflows"
        }
      }
    }
  }
}
```

Text S4: Audit and provenance records

CBIcall records structured audit artifacts (evidences) for each execution. The log.json file captures the resolved user parameters, selected workflow backend and provider, runtime context, and resource-bundle validation. The run-report.json file summarizes the completed run, including workflow identity, execution-contract fingerprints, resource identity, output inventories, and normalized final-output hashes. These records can be rendered as an optional static HTML report and compared across independent executions with cbicall compare-runs.

Excerpt 1. Resolved execution parameters and resource identity in log.json

```
{
  "param": {
    "mode": "single",
    "pipeline": "wes",
    "workflow_provider": "cbicall-core",
    "workflow_backend": "bash",
    "software_stack": "gatk-4.6",
    "genome": "b37",
    "resource": "cbicall-germline-resources-v1"
  },
  "config": {
    "version": "1.2.0",
    "profile": "local",
    "workflow": {
      "provider": "cbicall-core",
      "backend": "bash",
      "pipeline": "wes",
      "mode": "single",
      "software_stack": "gatk-4.6"
    },
    "resources": {
      "bundle": {
        "key": "cbicall-germline-resources-v1",
        "version": "v1",
        "compatible": true,
        "runtime_check": { "status": "verified" }
      }
    }
  }
}
```

Excerpt 2. Workflow and output fingerprints in run-report.json

```
{
  "framework": {
    "name": "CBIcall",
    "version": "1.2.0"
  },
  "execution_contract": {
    "provider": "cbicall-core",
    "backend": "bash",
    "workflow_key": "bash/wes/single/gatk-4.6/v1",
    "sha256": "b99c69173c29e454be25510a6026483fc22d9ffa73bf4a9760e9c410ca7ead4e",
    "normalized_command_sha256":
"f044a7fb18ded5b5938b0d2b1a6612c76a804c1a9db88aec66bdce058e980785"
  },
  "outputs": {
    "file_inventory": {
      "entries": 24,
```

```
      "total_bytes": 55767675,
      "sha256": "9609fa5fcbfdadbd12aee80baf041f57dc5f99fa51546b302de5cdcf5ffd7537"
    },
    "vcf_hash_reports": [
      {
        "file": "02_varcall/CNAG99901P.hc.QC.vcf.gz",
        "raw_sha256": "319161e94a5b154c7051c5442652ac8440b466cb47d3959bf3e11eda4378410d",
        "normalized_sha256": "621373dda8e79602e84f24b5e5082914b046c341ba64c7e15c8ce97172559baf",
        "normalized_records": "6"
      }
    ]
  }
}
```


Excerpt 3. Cross-run audit with cbicall compare-runs

```
Run comparison
  Runs             => 4
  Baseline         => ws5/run-report.json
  Compared         => hpc/run-report.json, gcloud/run-report.json, ws1/run-report.json

Framework
  CBIcall ver      => same
  Python ver       => same
  Java ver         => same
  Backend ver      => same

Pipeline
  Workflow key     => same
  Workflow hash    => same

Resources
  Resource key     => same
  Resource hash    => same

Outputs
  File inventory   => different
  VCF calls hash   => same
  VCF strict hash  => different: ws1/run-report.json
```

In this example, CBIcall distinguishes expected environment-specific differences in execution artifacts from reproducible final variant calls. The call-level VCF hash was identical across environments, whereas the strict full-record hash detected a minor ARM64-specific difference in non-call fields.

Table S1: Resource bundle "cbicall-germline-resources-v1" contents

Category	Resource	Version / Build	Archive path	Purpose	Reference
Reference genome	Human genome	hg38	Databases/genomes/hg38	Reference genome used for alignment and variant calling	Genome Reference Consortium
Annotation database	dbSNP	b144 (GRCh37)	Databases/dbSNP/human_9606_b144_GRCh37p13	Known variants used for annotation and recalibration	(Sherry *et al.* 2001)
Annotation database	dbSNP	b146 (GRCh38)	Databases/dbSNP/human_9606_b146_GRCh38p2	Known variants annotation and recalibration	(Sherry *et al.* 2001)
Workflow resources	GATK resource bundle	—	Databases/GATK_bundle	Known-sites resources required by GATK workflows	Broad Institute
Capture resources	Agilent SureSelect	—	Databases/Agilent_SureSelect	Target interval definitions used for exome sequencing analyses	Agilent Technologies
Mitochondrial resources	mtDNA references	—	Databases/mtDNA	Mitochondrial reference sequences (rCRS and RSRS) and derived hg19-based FASTA files (chrM, hg19RCRS, hg19RSRS), compressed FASTA resources, and GMAP alignment databases	(Calabrese *et al.* 2014)
Alignment software	BWA	0.7.18	NGSutils/bwa-0.7.18*	Read alignment to the reference genome	(Li Heng and Durbin 2009, Li Heng 2013)
Variant analysis framework	GATK	3.5; 4.6	NGSutils/gatk/	Germline variant calling and preprocessing workflows	(McKenna *et al.* 2010)
BAM processing utilities	SAMtools	0.1.19; 1.3	NGSutils/samtools-*	BAM/SAM manipulation; 1.3 is used by MToolBox	(Li H. *et al.* 2009)
BAM processing utilities	Picard	2.25	NGSutils/picard-2.25	Duplicate marking and BAM processing	Broad Institute
Genomic interval utilities	BEDTools	2.x	NGSutils/bedtools2*	Genomic interval and feature manipulation	(Quinlan and Hall 2010)
Runtime dependency	Java	8	NGSutils/java8	Java runtime required by GATK and Picard	Oracle
Runtime dependency	Python	2.7 (portable)	NGSutils/python_2.7/linux-x86_64/python27_portable	Legacy runtime required by specific pipeline components	Python Software Foundation
mtDNA analysis pipeline	MToolBox	1	NGSutils/MToolBox-master	Pipeline for mitochondrial genome assembly and heteroplasmy analysis	(Calabrese *et al.* 2014)

* Indicates directories containing architecture-specific builds (e.g., x86_64 and ARM64).

CBIcall v1.2.0 comes with a resource bundle (cbicall-germline-resources-v1") containing the reference datasets, auxiliary files, and third-party bioinformatics utilities required by the validated workflows. The main components included in this bundle are summarized in the table above.

Table S2. Benchmarking against GIAB truth set

Table S2. Summary statistics from the GIAB trio benchmark.

Sample	Metric	SNP ALL	SNP PASS	INDEL ALL	INDEL PASS
HG002	F1	99.15%	94.93%	98.57%	95.71%
	Recall	99.27%	90.41%	98.46%	92.49%
	Precision	99.02%	99.91%	98.68%	99.15%
HG003	F1	99.13%	94.93%	98.60%	95.47%
	Recall	99.28%	90.44%	98.55%	92.07%
	Precision	98.98%	99.90%	98.64%	99.13%
HG004	F1	99.12%	94.91%	98.52%	95.39%
	Recall	99.27%	90.40%	98.45%	91.98%
	Precision	98.98%	99.90%	98.59%	99.07%

Text S6. Cross-environment reproducibility assessment

To assess portability and reproducibility across computing environments, CBIcall was executed using the same user parameters YAML, workflow definition, software stack, and resource bundle on four independent systems: a Google Cloud Ubuntu 22.04 virtual machine (gcloud), an x86_64 Linux Mint workstation (ws5), an ARM64 Ubuntu 24.04 virtual machine running on macOS hardware (ws1), and an x86_64 CentOS 7.9 SLURM-managed HPC cluster (hpc).

Completed runs were compared using cbicall compare-runs, which evaluates recorded audit artifacts including workflow identity, resource identity, execution metadata, output inventories, and VCF fingerprints. Final WES VCFs were compared using two complementary hashes: a call-level fingerprint based on CHROM, POS, REF, ALT, FILTER, and sample genotype fields, and a stricter full-record fingerprint that also captures QUAL, INFO, FORMAT fields, annotations, and numerical values.

The call-level VCF fingerprint was identical across all four environments, indicating reproducible final variant calls. The stricter full-record fingerprint also matched across the x86_64 workstation, Google Cloud, and HPC runs, while the ARM64 run showed a minor strict-record difference without altering the call-level fingerprint. This pattern is consistent with architecture-dependent numerical or formatting drift in non-call fields while preserving the reported variant calls. A representative compare-runs output excerpt is shown below.

Comparison target	VCF calls fingerprint	VCF strict records fingerprint	Interpretation
ws5 baseline	727f877de6ec...0a26a976	a659e0105d8b...4a5539c0	Baseline.
hpc	727f877de6ec...0a26a976	a659e0105d8b...4a5539c0	Matches baseline at call and strict-record level.
gcloud	727f877de6ec...0a26a976	a659e0105d8b...4a5539c0	Matches baseline at call and strict-record level.
ws1	727f877de6ec...0a26a976	5c03b2a73c25...7407487e	Call-equivalent to baseline; strict-record-only numeric drift.

Text S7: Variant filtering

For single-sample analyses, records in each final QC VCF file (QC.vcf.gz) were first restricted to FILTER=PASS in a separate preceding step. Genotypes with sequencing depth (DP) < 10 or genotype quality (GQ) < 20 were then set to missing, and only biallelic SNPs were retained using bcftools (Danecek *et al.* 2021):

```
bcftools filter -e 'FMT/DP<10 || FMT/GQ<20' -S . -Ou input.vcf.gz |
bcftools view -m2 -M2 -v snps -Oz -o output.clean.vcf.gz
```

The filtered single-samples from each cohort were subsequently merged into cohort-level VCF files using:

```
bcftools merge -Oz -o merged.vcf.gz -l sample_list.txt
```

For joint genotyping, the same filtering procedure was applied directly to the multi-sample cohort VCF generated by the CBIcall WES cohort workflow.

Figure S1: dbGaP samples selection

The dbGaP dataset used in the CBIcall use case (accession phs001172.v1.p2) comprised 618 samples. As an example of population-structure quality control, Principal Component Analysis (PCA) was performed using PASS variants generated by CBIcall and converted to PLINK format (Purcell *et al.* 2007). Samples were coloured according to self-reported ancestry and sex.

a

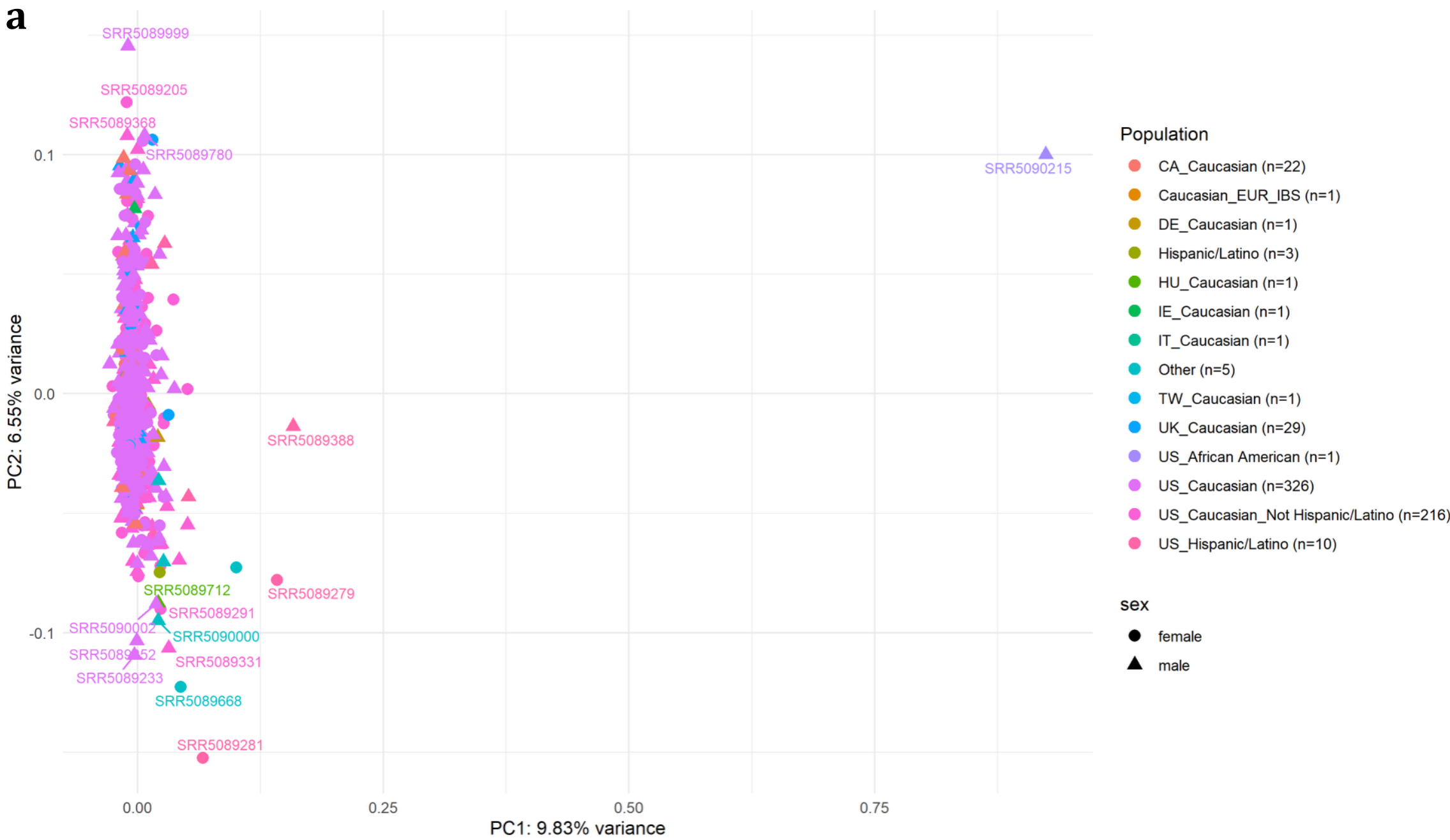

Figure S1a. Initial PCA of 618 samples.

The initial PCA (Figure S1a) identified one sample (SRR5090215) reported with US_African_American ancestry that clustered separately from the main cohort. That sample was excluded and the PCA was repeated (Figure S1b).

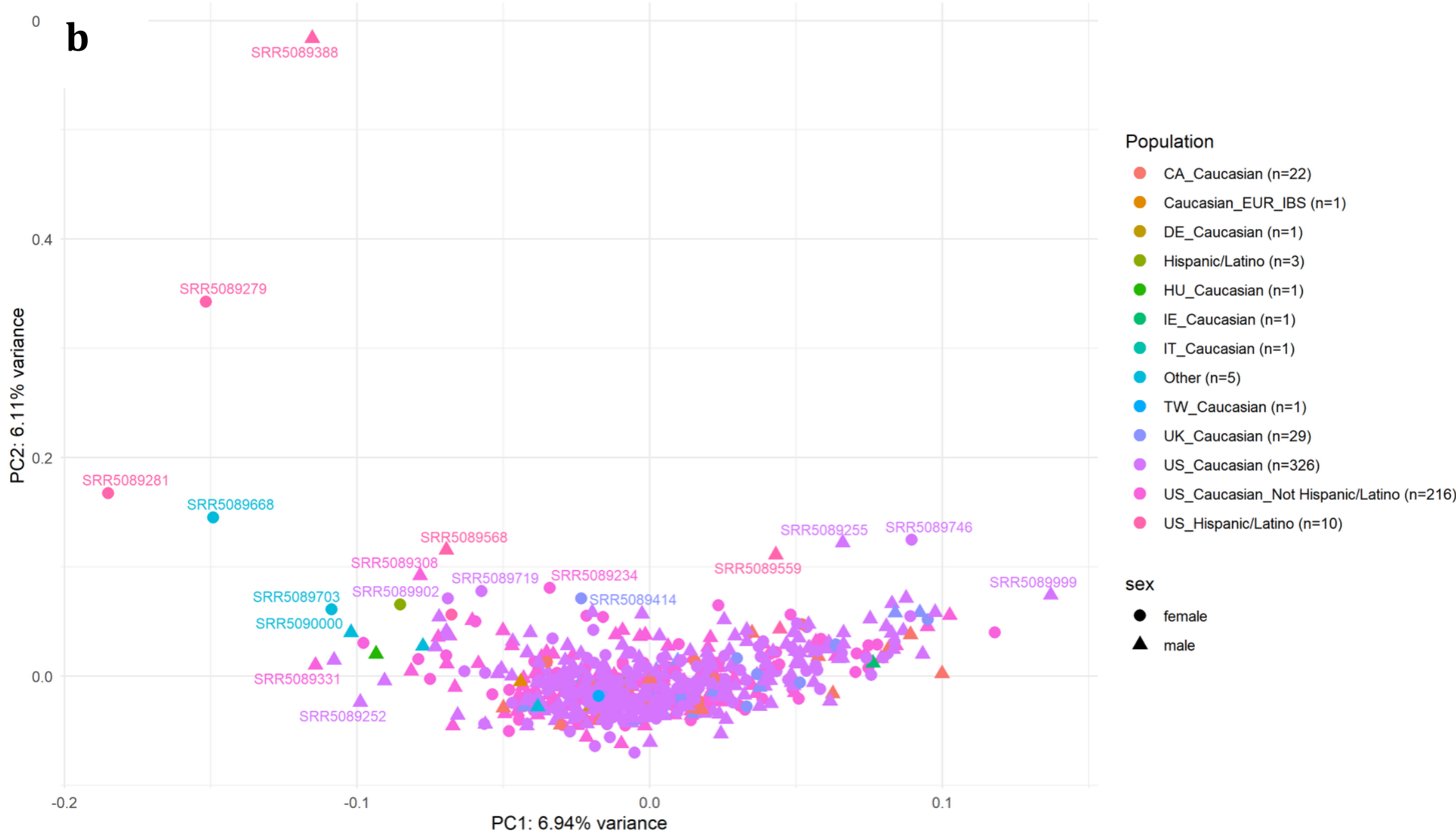

Figure S1b. PCA after removal of sample SRR5090215 (n=617).

The second PCA identified additional self-reported Hispanic/Latino samples (e.g., SRR5089388) that remained distinct from the main cluster. To obtain a more genetically homogeneous cohort, all self-reported Hispanic/Latino samples, together with samples classified as “Other”, were excluded from subsequent analyses. The complete list of excluded samples is provided in the table below:

**Run**	**Sex**	**Self-reported ancestry**
SRR5089348	male	Hispanic/Latino
SRR5089349	male	Hispanic/Latino
SRR5089568	male	US_Hispanic/Latino
SRR5089668	female	Other (US More than 1 race)
SRR5089698	male	Other (IN Asian)
SRR5089703	female	Other (BR Other)
SRR5090000	male	Other (IN Asian)
SRR5090163	male	US_Hispanic/Latino
SRR5090210	male	Other (US American Indian)
SRR5089347	female	Hispanic/Latino
SRR5089354	male	US_Hispanic/Latino
SRR5089361	female	US_Hispanic/Latino
SRR5089388	male	US_Hispanic/Latino
SRR5089424	male	US_Hispanic/Latino
SRR5089515	female	US_Hispanic/Latino
SRR5089559	male	US_Hispanic/Latino
SRR5089279	female	US_Hispanic/Latino

SRR5089281	female	US_Hispanic/Latino

After removal of these samples, PCA was repeated on the remaining 599 individuals (Figure S1c), resulting in a more homogeneous cluster.

c

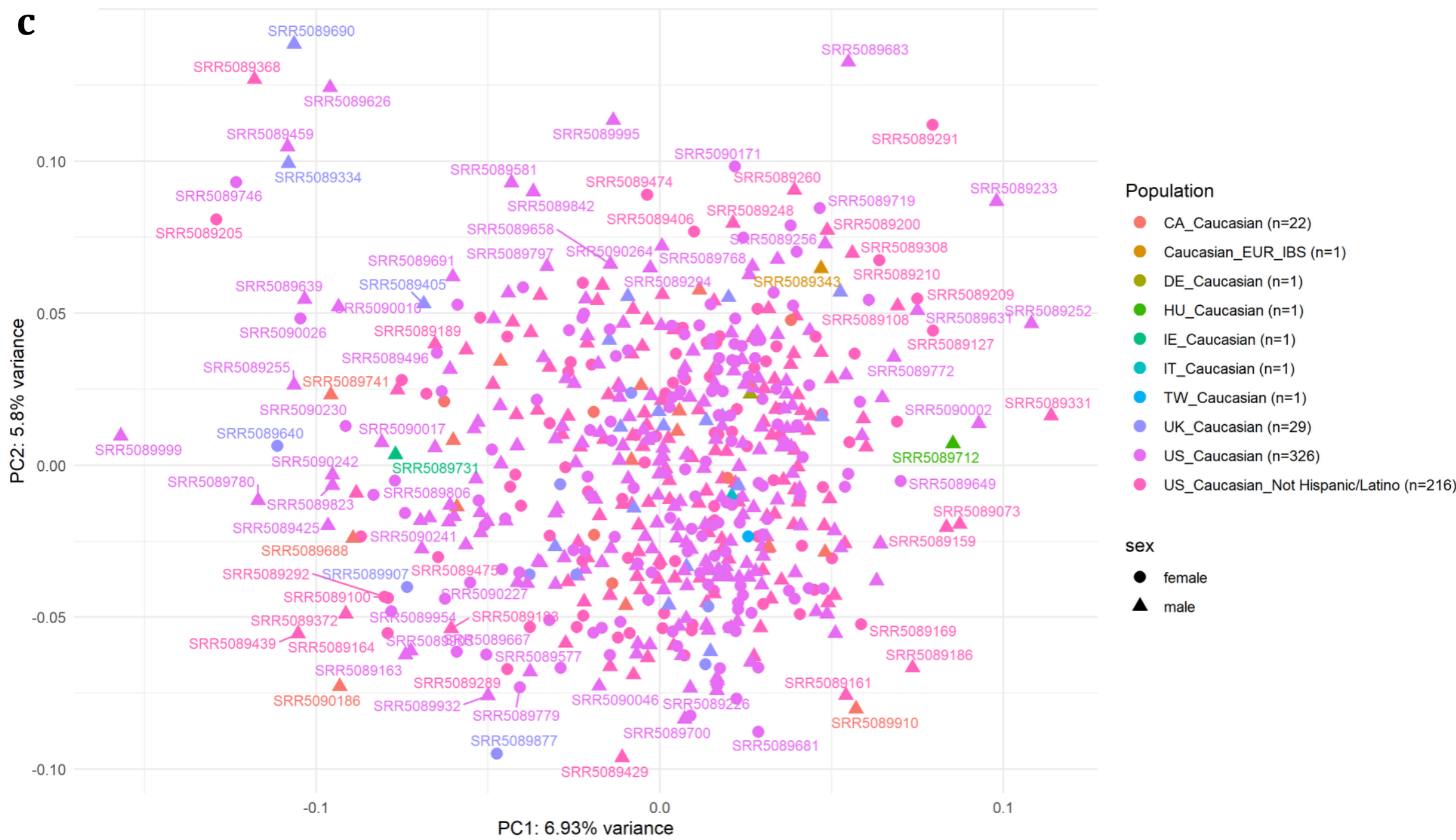

Figure S1c. PCA after removal of 19 samples by self-reported ancestry (n=599).

Figure S2: Principal Component Analysis of cases and controls.

As a preliminary quality-control assessment, we performed PCA using genotypes obtained from the single-sample variant-calling workflow. To ensure that PCA was performed on a common set of markers, only variant sites present in both cohorts were retained using bcftools isec:

```
bcftools isec -p isec_out \
    1000G.PASS.clean.merged.vcf.gz \
    phs001172.PASS.clean.merged.vcf.gz
```

The resulting shared variant set was converted to PLINK format for PCA analysis.

a

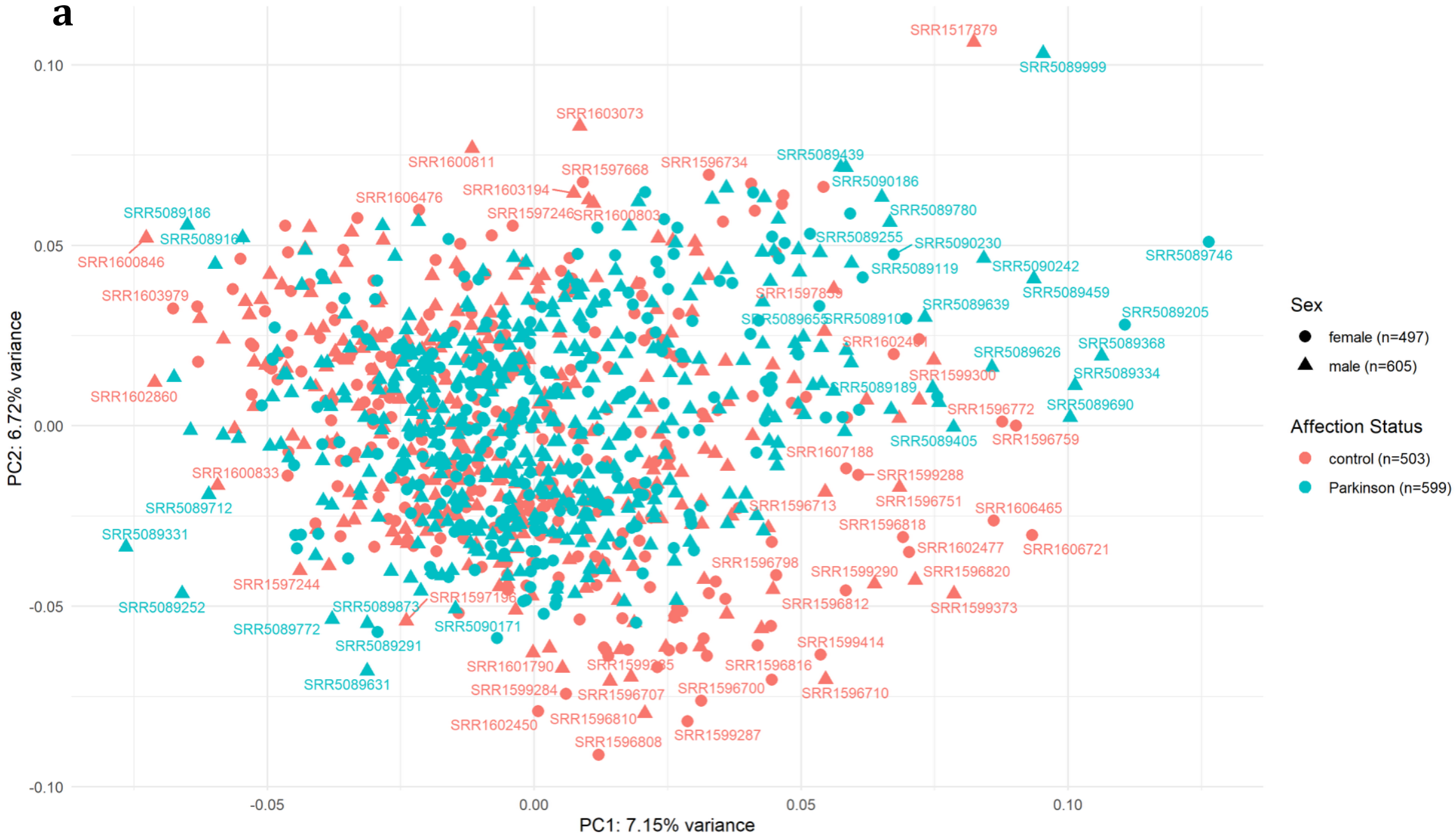

Figure S2a. Principal component analysis (PCA) based on genotypes derived from CBIcall variant calls (WES, single-sample mode) and converted to PLINK format. The analysis used the intersection of variants between Parkinson's disease cases from dbGaP (accession phs001172.v1.p2; n = 599) and controls from the 1000 Genomes Project (n = 503).

The final dataset comprised 503 controls and 599 cases. Reported sex distribution was:

	Control	Parkinson
**Female**	263	234
**Male**	240	365

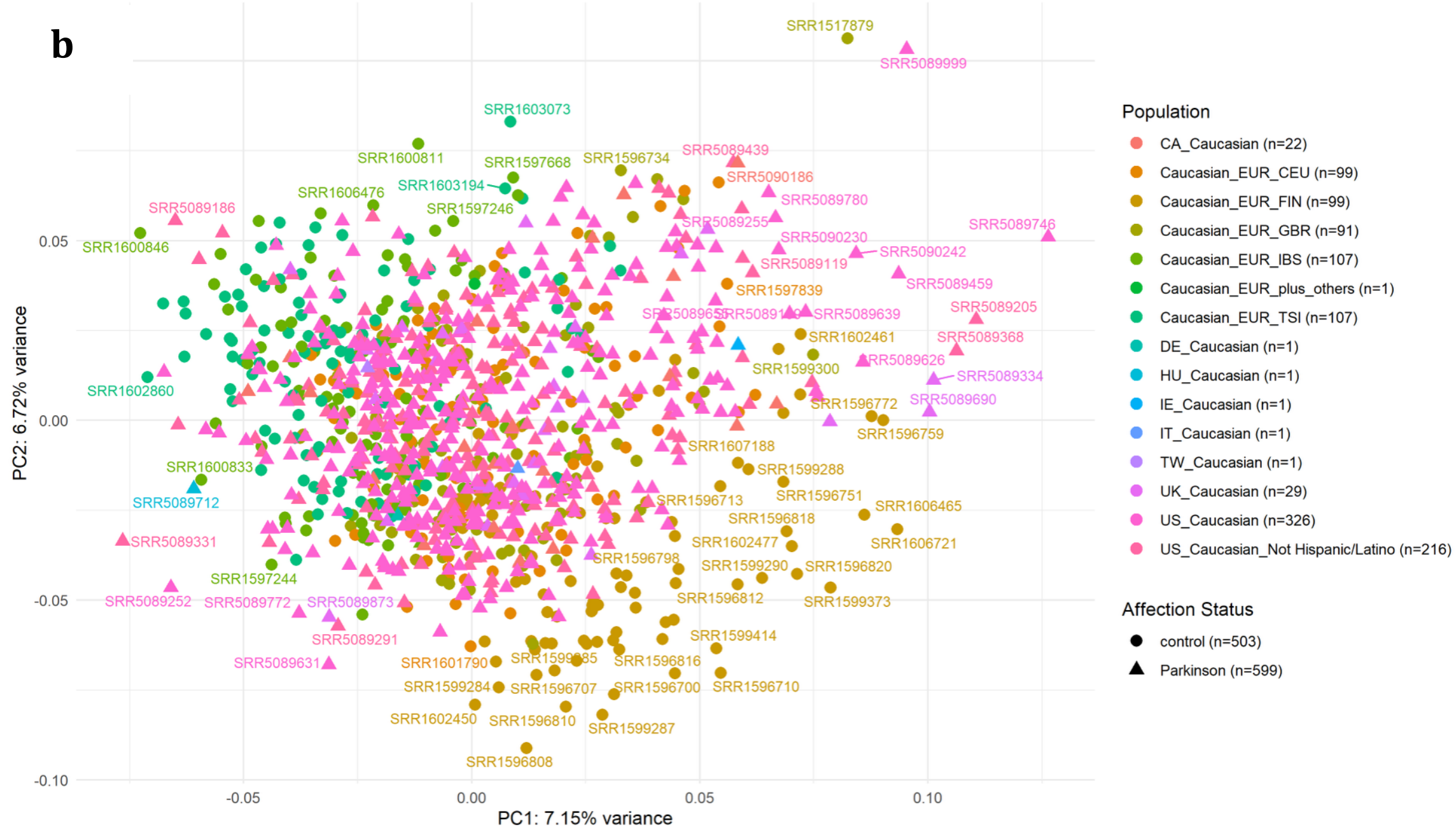

Figure S2b. Principal component analysis (PCA) based on the same genotype dataset shown in Figure S2a, with samples coloured according to self-reported ethnicity.

Figure S3: Distribution of variant-site read depth (DP) across analysis strategies.

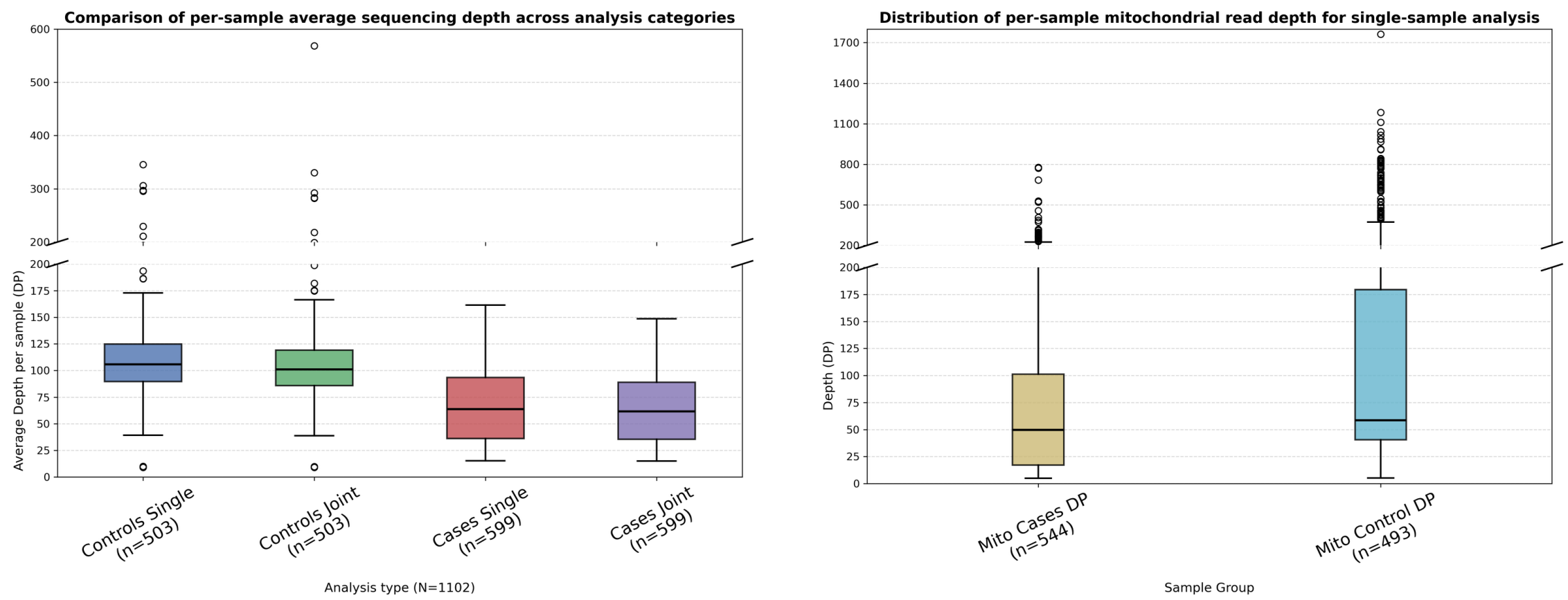

Figure S3. **Left**. Distribution of average variant-site read depth (DP) per sample, grouped by analysis type for single vs joint analysis. **Right**. Distribution of average DP for mitochondrial single-sample analysis. A broken y-axis is applied to enhance visualization of inter-group variability (0–200, step 25; 200–600, step 100, left; 200–1700, step 300, right). Boxplots show that DP values are comparable across analysis strategies, indicating consistent per-sample coverage and that DP is greater in Control samples.

Boxplots of variant-site read depth (DP) per sample across analysis strategies are shown in Figure S3. For WES analyses, DP distributions were highly comparable between single-sample and joint genotyping modes. The 1000 Genomes cohort showed a median per-sample DP of 106× for single-sample analysis and 101× for joint calling, whereas the phs001172 cohort showed a median per-sample DP of 64× for single-sample analysis and 61× for joint calling.

For mitochondrial DNA analysis, median per-sample DP was 59× for controls and 50× for cases. Thus, DP fell within the range generally considered sufficient for reliable variant detection (see also Figure S4).

Table S3. Variant filtering statistics for single-sample and joint genotyping analyses

Table S3. Summary of variant counts before and after quality filtering (FILTER=PASS) in single-sample and joint genotyping analyses. The table reports the number of variants before filtering, the number retained after filtering, and the absolute and relative number of variants removed. Percentages indicate the proportion of variants discarded relative to the unfiltered dataset.

Dataset	All variants	PASS variants	Variants removed	% removed
**Single (total)**	1,242,805	648,391	594,414	**47.83%**
└─ Single-only	526,197	221,271	304,926	**57.95%**
**Joint (total)**	742,055	441,433	300,622	**40.51%**
└─ Joint-only	25,447	14,313	— (increase)	—

The single-sample dataset decreased from 1,242,805 to 648,391 variants after filtering, corresponding to a loss of 47.83% of calls. In contrast, the joint-genotyped dataset decreased from 742,055 to 441,433 variants, representing a reduction of 40.51%. Notably, variants exclusive to the single-sample analysis exhibited the highest proportional reduction, with 57.95% being removed after filtering, indicating that a large fraction of these calls correspond to low-confidence variants. These results demonstrate that joint genotyping produces a more conservative and higher-quality variant set, whereas single-sample calling generates a larger number of variants but includes a higher proportion of low-confidence calls that are subsequently removed by quality filtering.

Table S4. Summary statistics of variant counts per sample across cohorts, variant classes, and genotyping strategies

Table S4. Summary statistics of variant counts per sample across sequencing data types (WES or mtDNA), variant classes (SNVs or INDELs), cohorts (phs001172 or 1000 Genomes Project), and genotyping strategies (single-sample or joint calling). For each combination, the table reports the number of samples analyzed and the distribution of variant counts per VCF file (minimum, first quartile (Q1), median, third quartile (Q3), and maximum). These statistics were used to assess cohort comparability and the impact of genotyping strategy on variant discovery.

Sequencing Data	Genotyping Strategy	Variant Type	Cohort	# samples	Min # variants in vcf	Q1	Median	Q3	Max # variants in vcf
WES	Single-sample	SNVs	1000G	503	15,984	19,714	22,338	23,515	30,704
WES	Joint	SNVs	1000G	503	14,695	19,901	22,447	23,760	30,890
WES	Single-sample	INDELs	1000G	503	415	514	578	631	891
WES	Joint	INDELs	1000G	503	465	656	722	803	1,167
mtDNA	NA	SNVs	1000G	493	1	3,313	3,911	4,143	4,725
mtDNA	NA	INDELs	1000G	493	0	40	43	52	84
WES	Single-sample	SNVs	phs001172	599	15,509	21,914	22,502	23,086	64,401*
WES	Joint	SNVs	phs001172	599	15,632	21,868	22,622	23,335	38,263*
WES	Single-sample	INDELs	phs001172	599	261	502	595	669	2,309
WES	Joint	INDELs	phs001172	599	337	633	760	857	1,628
mtDNA	NA	SNVs	phs001172	544	1	1,898	2,865	4,219	4,793
mtDNA	NA	INDELs	phs001172	544	0	26	40	49	113

* The maximum values observed (30k~64k variants) correspond to a small number of outlier samples (Sample SRR5090246), whereas the median variant counts remain highly consistent across cohorts.
Note: mtDNA sample numbers differ from the initial cohort, as a subset of samples did not meet the MToolBox coverage threshold required for reliable variant calling (493 for 1000G; 544 for dbGaP).

For WES single-sample SNVs, both cohorts exhibited nearly identical median values (22,502 for phs001172 and 22,338 for 1000 Genomes (1000G)), with highly overlapping interquartile ranges, indicating comparable variant discovery rates and sequencing depth. A similar pattern was observed for WES INDELs, with medians of 595 and 578 variants per sample for phs001172 and 1000 Genomes, respectively.

Mitochondrial variant counts also showed broadly comparable distributions between cohorts, although modest differences were observed, particularly for SNVs, where the median number of variants was slightly higher in the 1000 Genomes cohort (3,911) than in phs001172 (2,865). These differences are likely attributable to technical factors such as

variable mitochondrial coverage in WES capture protocols and differences in sequencing depth rather than biological divergence.

We further evaluated the impact of genotyping strategy on variant discovery by comparing single-sample and joint-calling approaches. For WES SNVs, the median number of variants per sample obtained by joint calling (22,447 for 1000G and 22,622 for phs001172) was highly consistent with those observed in the single-sample analyses of the respective cohorts. A similar trend was observed for INDELs, with joint-calling medians (722 for 1000G and 760 for phs001172) falling within the interquartile ranges of both single-sample datasets.

Overall, the similarity in variant count distributions across cohorts and analysis strategies supports their use in this representative CBIcall deployment analysis.

Table S5. Transition/transversion (Ti/Tv) and heterozygous/homozygous (Het/Hom) ratios for the single-sample and joint-genotyping callsets.

Table S5. Summary statistics of for both cohorts (phs001172 or 1000 Genomes Project), and genotyping strategies (single-sample or joint calling).

Callset	Transitions (Ti)	Transversions (Tv)	Ti/Tv	Mean Het/Hom	Median Het/Hom
SINGLE	334,584	180,299	1.86	1.6788	1.6622
JOINT	286,435	115,241	2.49	1.5878	1.5894

To address the reviewers' request for additional variant-quality metrics, we calculated transition/transversion (Ti/Tv) and heterozygous/homozygous (Het/Hom) ratios for both callsets. For the single-sample analysis, per-sample VCF files were filtered and merged using the same strategy described in Text S7. To ensure comparability between callsets, analyses were restricted to PASS variants located on the autosomes (chromosomes 1–22).

After filtering, the single-sample callset contained 514,883 variants, whereas the joint-genotyped callset contained 401,676 variants. Ti/Tv ratios were calculated using bcftools stats, and Het/Hom ratios were derived from genotype counts obtained with bcftools query and standard Unix command-line utilities.

The single-sample strategy identified 113,207 additional SNPs compared with the joint-genotyped callset. However, these additional variants were enriched for transversions, resulting in a substantially lower Ti/Tv ratio (1.86 versus 2.49). Because true germline variants are expected to be enriched for transitions, the higher Ti/Tv ratio observed after joint genotyping is consistent with the removal of lower-confidence variant calls during cohort-level genotype refinement.

Het/Hom ratios were broadly comparable between callsets (mean 1.68 versus 1.59; median 1.66 versus 1.59), indicating that genotype composition remained largely consistent despite differences in the number of retained variants.

Figure S4: Mitochondrial variant calling and filtering

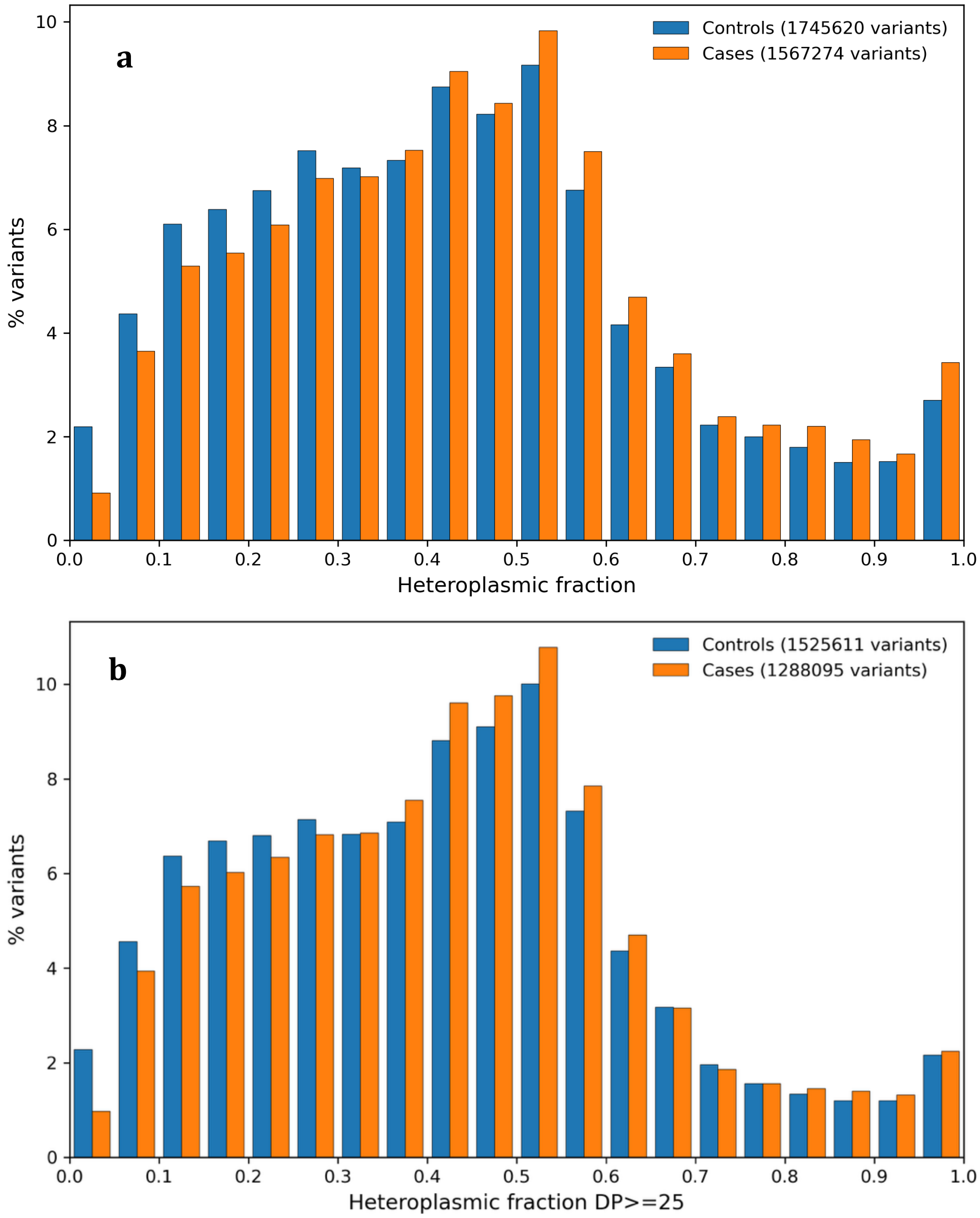

Figure S4. Distribution of heteroplasmic mitochondrial variants in controls and cases. (A) Distribution of heteroplasmic variants before applying a sequencing depth filter. (B) Distribution after filtering variants to a sequencing depth (DP) ≥ 25. Variants were further restricted to Heteroplasmic Fraction (HF) > 0.3 to focus on moderate- to high-level heteroplasmies, which have greater potential functional relevance than low-level heteroplasmies of uncertain biological significance. Together with a minimum sequencing depth of 25×, this conservative filter also minimized the contribution of low-frequency technical artefacts.

Figure S5: Relationship between mitochondrial locus length and heteroplasmic variant frequency

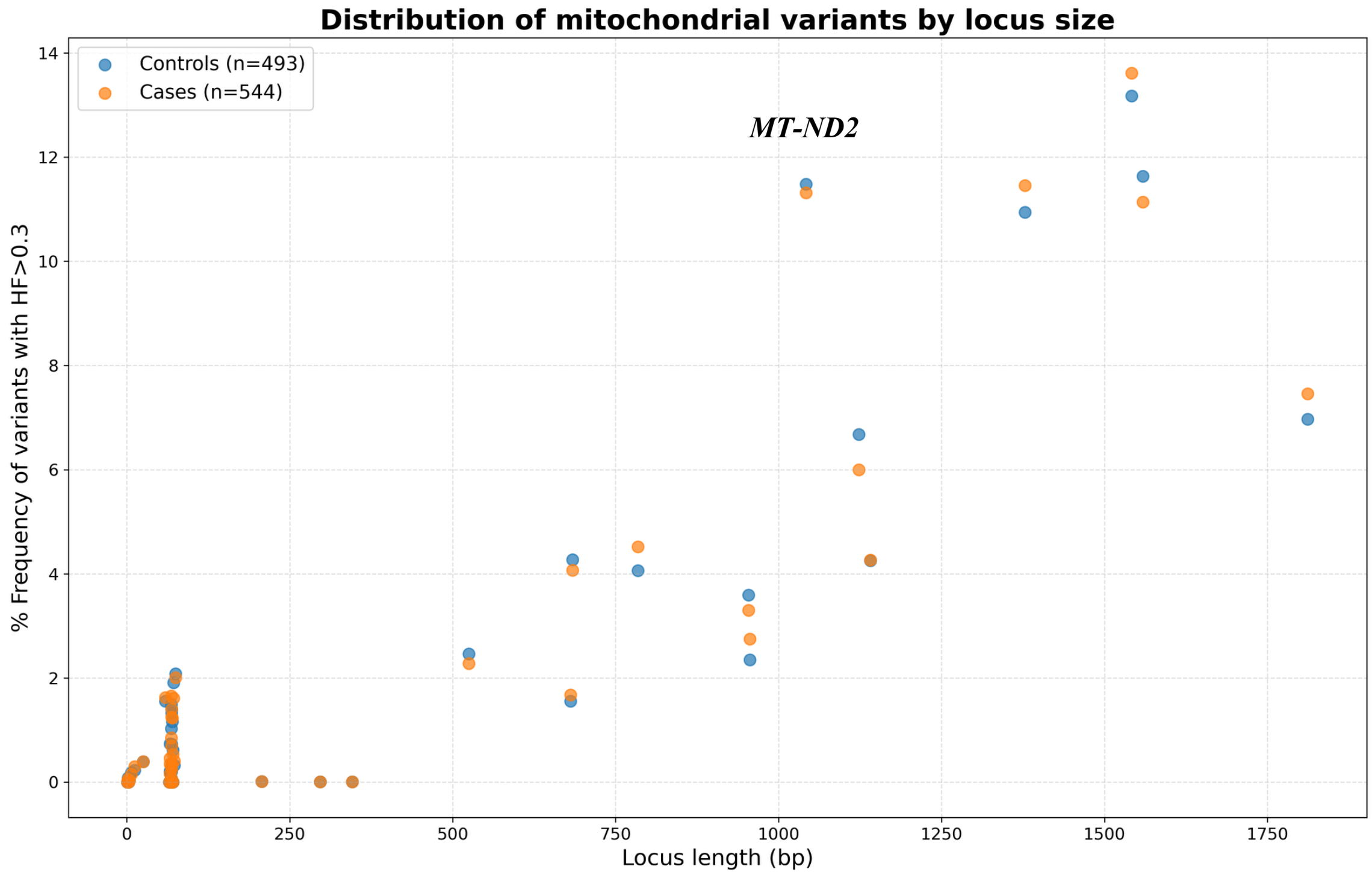

Figure S5. Scatter plot showing the relationship between mitochondrial locus length and the % frequency of heteroplasmic variants (HF > 0.3) across the case and control cohorts.

A detailed analysis of heteroplasmic variant distribution across mitochondrial loci revealed consistent increasing trend with locus size, consistent with previous observations.

Despite differences in sequencing strategies between cohorts, both case and control datasets exhibited similar patterns of heteroplasmic variation. A small number of short mitochondrial loci showed relatively higher variability in heteroplasmic variant counts. *MT-ND2* also displayed an increased number of variants. An explanation for these deviations could be influenced by technical factors such as variable mitochondrial coverage in whole-exome sequencing data and increased stochastic effects in short genomic intervals.

Figure S6: Example mtDNA HTML report

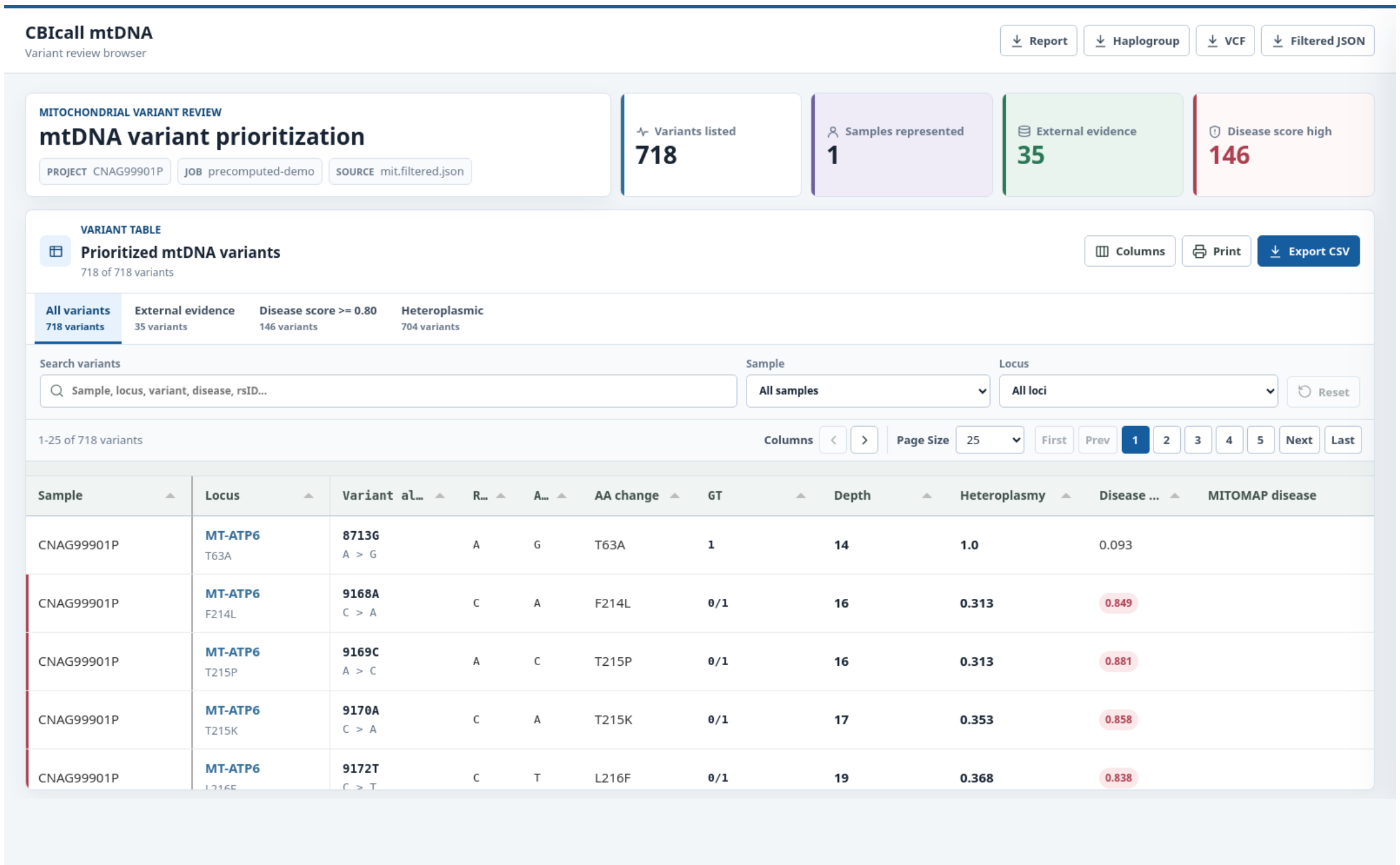

Figure S6. The Download menu provides access to four key analysis outputs. The Report (*mit_prioritized_variants.txt*) is a tab-separated file containing MToolBox-prioritized variant annotations augmented by CBIcall with the REF, ALT, GT, DP, and HF fields. The Haplogroup (*mt_classification_best_results.csv*) is a comma-separated file reporting the best predicted mitochondrial haplogroup(s). The VCF (*VCF_file.vcf*) contains the MToolBox variant calls together with per-sample genotype, read depth, heteroplasmy fraction, and heteroplasmy confidence intervals. The Filtered JSON (*mit.filtered.json*) contains the subset of prioritized variants retained by the browser filters.

The interactive table displays the prioritized mitochondrial variants retained after filtering. **Sample** identifies the analyzed sample, and **Locus** indicates the mitochondrial gene or annotated region. **Variant allele** combines the mitochondrial position with the alternative allele. **Ref** and **Alt** report the RSRS reference allele and the detected alternative allele(s), respectively. **AA change** reports the predicted amino acid substitutions for protein-coding variants. **GT** follows the VCF genotype convention, where 0 denotes the reference allele and values ≥ 1 correspond to the alternative allele(s) listed in **Alt**. **Depth** reports the read depth at the variant position, and **Heteroplasmy** reports the estimated alternative allele fraction. The corresponding lower and upper confidence limits are provided as **CILOW** and **CIUP** in the downloadable VCF. Definitions of the remaining annotation fields are available in the MToolBox documentation.

References

Calabrese C, Simone D, Diroma MA *et al.* MToolBox: a highly automated pipeline for heteroplasmy annotation and prioritization analysis of human mitochondrial variants in high-throughput sequencing. *Bioinformatics* 2014;**30**(21):3115–7. https://doi.org/10.1093/bioinformatics/btu483.

Danecek P, Bonfield JK, Liddle J *et al.* Twelve years of SAMtools and BCFtools. *GigaScience* 2021;**10**(2):giab008. https://doi.org/10.1093/gigascience/giab008.

Li H., Handsaker B, Wysoker A *et al.* The Sequence Alignment/Map format and SAMtools. *Bioinformatics* 2009;**25**(16):2078–9. https://doi.org/10.1093/bioinformatics/btp352.

Li Heng. Aligning sequence reads, clone sequences and assembly contigs with BWA-MEM, arXiv:1303.3997. Preprint, arXiv, 26 May 2013. https://doi.org/10.48550/arXiv.1303.3997.

Li Heng, Durbin R. Fast and accurate short read alignment with Burrows-Wheeler transform. *Bioinformatics* (Oxford, England) 2009;**25**(14):1754–60. https://doi.org/10.1093/bioinformatics/btp324.

McKenna A, Hanna M, Banks E *et al.* The Genome Analysis Toolkit: a MapReduce framework for analyzing next-generation DNA sequencing data. *Genome Res* 2010;**20**(9):1297–303. https://doi.org/10.1101/gr.107524.110.

Purcell S, Neale B, Todd-Brown K *et al.* PLINK: a tool set for whole-genome association and population-based linkage analyses. *Am J Hum Genet* 2007;**81**(3):559–75. https://doi.org/10.1086/519795.

Quinlan AR, Hall IM. BEDTools: a flexible suite of utilities for comparing genomic features. *Bioinformatics* 2010;**26**(6):841–2. https://doi.org/10.1093/bioinformatics/btq033.

Sherry ST, Ward MH, Kholodov M *et al.* dbSNP: the NCBI database of genetic variation. *Nucleic Acids Res* 2001;**29**(1):308–11. https://doi.org/10.1093/nar/29.1.308.
